# Supplementary material for: A Novel Multi-Strain E3 Probiotic Formula Improved the Gastrointestinal Symptoms and Quality of Life in Chinese Psoriasis Patients
Source: Microorganisms. 2024 Jan 19;12(1):208. doi: 10.3390/microorganisms12010208 (PMC10820679; doi:10.3390/microorganisms12010208)
Supplement: Supplementary file 1 [file microorganisms-12-00208-s001.zip › Figures and Tables caption_20231221.pdf]

Supplementary Figure S1. Boxplots of alpha diversity of psoriasis patients at week 0 and week 8 based on (A) ACE Index, (B) Chao1 Index, (C) Faith's Phylogenetic Diversity, (D) the Observed OTUs, (E) Shannon Diversity Index, and (F) Simpson Index.

Supplementary Figure S2. Boxplots of alpha diversity of responder at week 0 and week 8 based on (A) ACE Index, (B) Chao1 Index, (C) Faith's Phylogenetic Diversity, (D) the Observed OTUs, (E) Shannon Diversity Index, and (F) Simpson Index.

Supplementary Figure S3. Boxplots of alpha diversity of non-responder at week 0 and week 8 based on (A) ACE Index, (B) Chao1 Index, (C) Faith's Phylogenetic Diversity, (D) the Observed OTUs, (E) Shannon Diversity Index, and (F) Simpson Index.

Supplementary Figure S4. Boxplots of alpha diversity of responder and non-responder at week 0 based on (A) ACE Index, (B) Chao1 Index, (C) Faith's Phylogenetic Diversity, (D) the Observed OTUs, (E) Shannon Diversity Index, and (F) Simpson Index.

Supplementary Table S1. ADONIS Results of Beta Diversity Distances.

Supplementary Table S2. Taxo classification of differentially abundant ASVs identified by ANCOM-BC2.

Supplementary Table S3. Differentially abundant ASVs (taxon assigned by q2-feature-classifier) identified by ANCOM-BC2 between responder and non-responder groups.
