# Supplementary material for: A Novel Multi-Strain E3 Probiotic Formula Improved the Gastrointestinal Symptoms and Quality of Life in Chinese Psoriasis Patients
Source: Microorganisms. 2024 Jan 19;12(1):208. doi: 10.3390/microorganisms12010208 (PMC10820679; doi:10.3390/microorganisms12010208)

Supplementary Figure S1

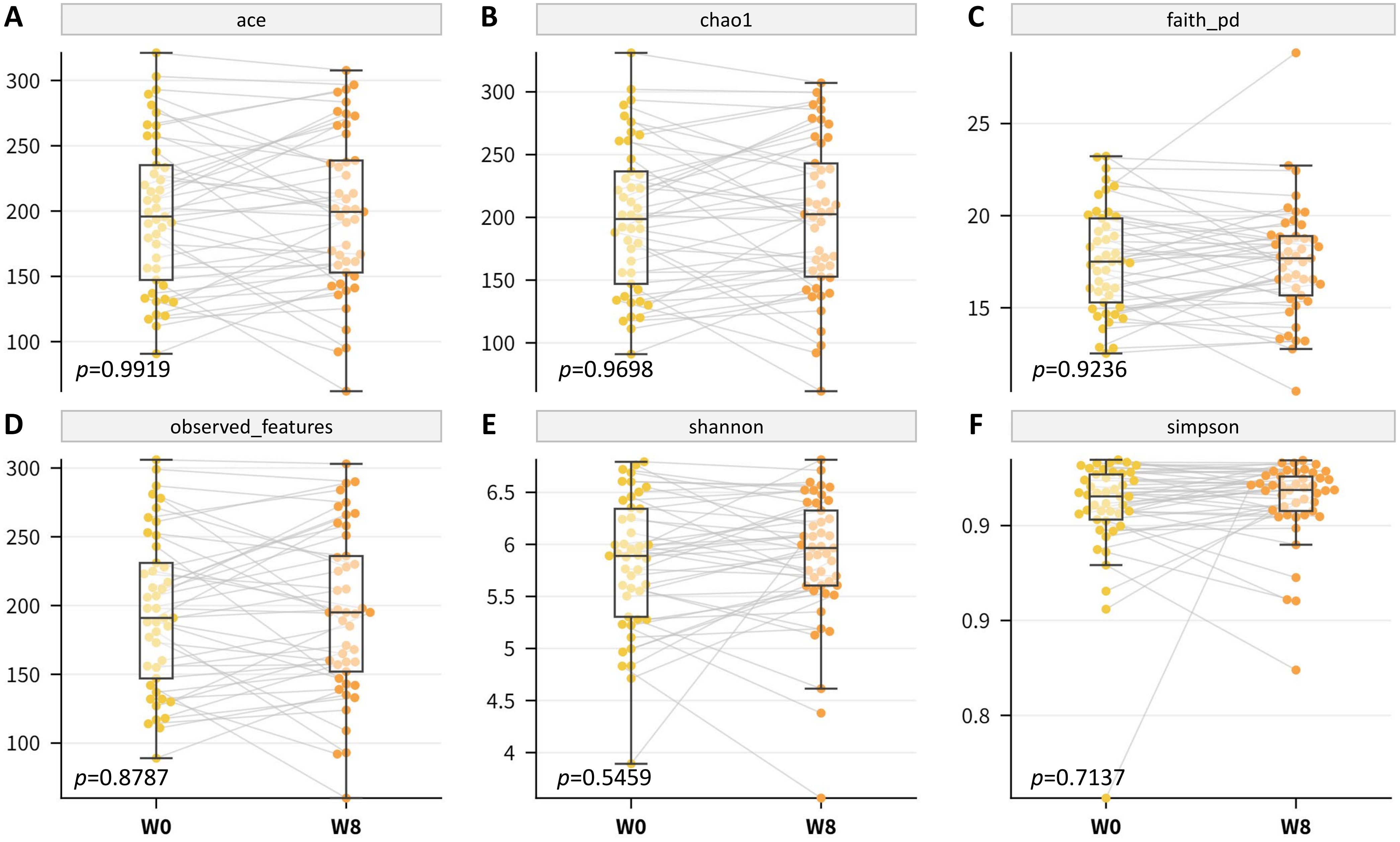

Supplementary Figure S2

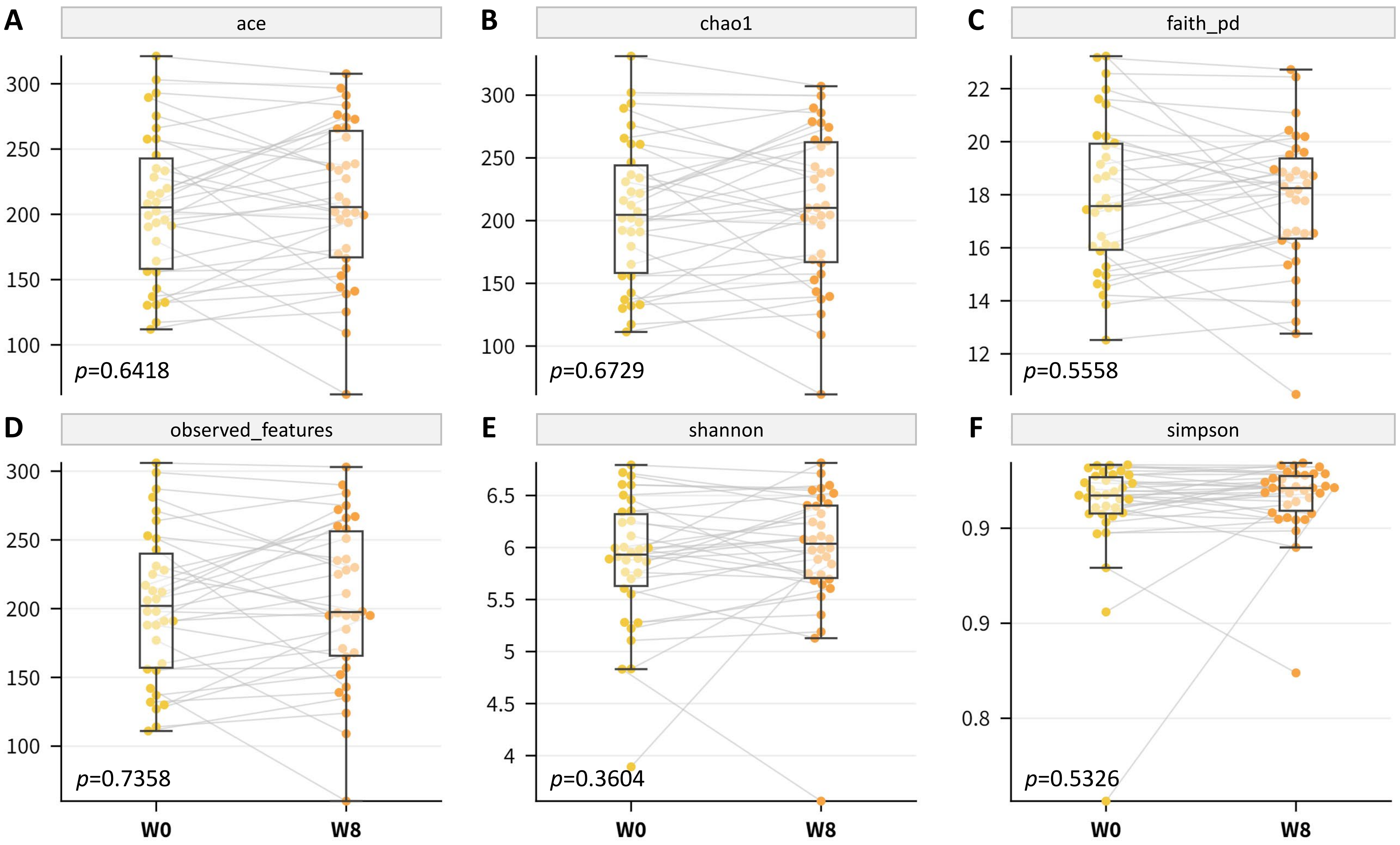

Supplementary Figure S3

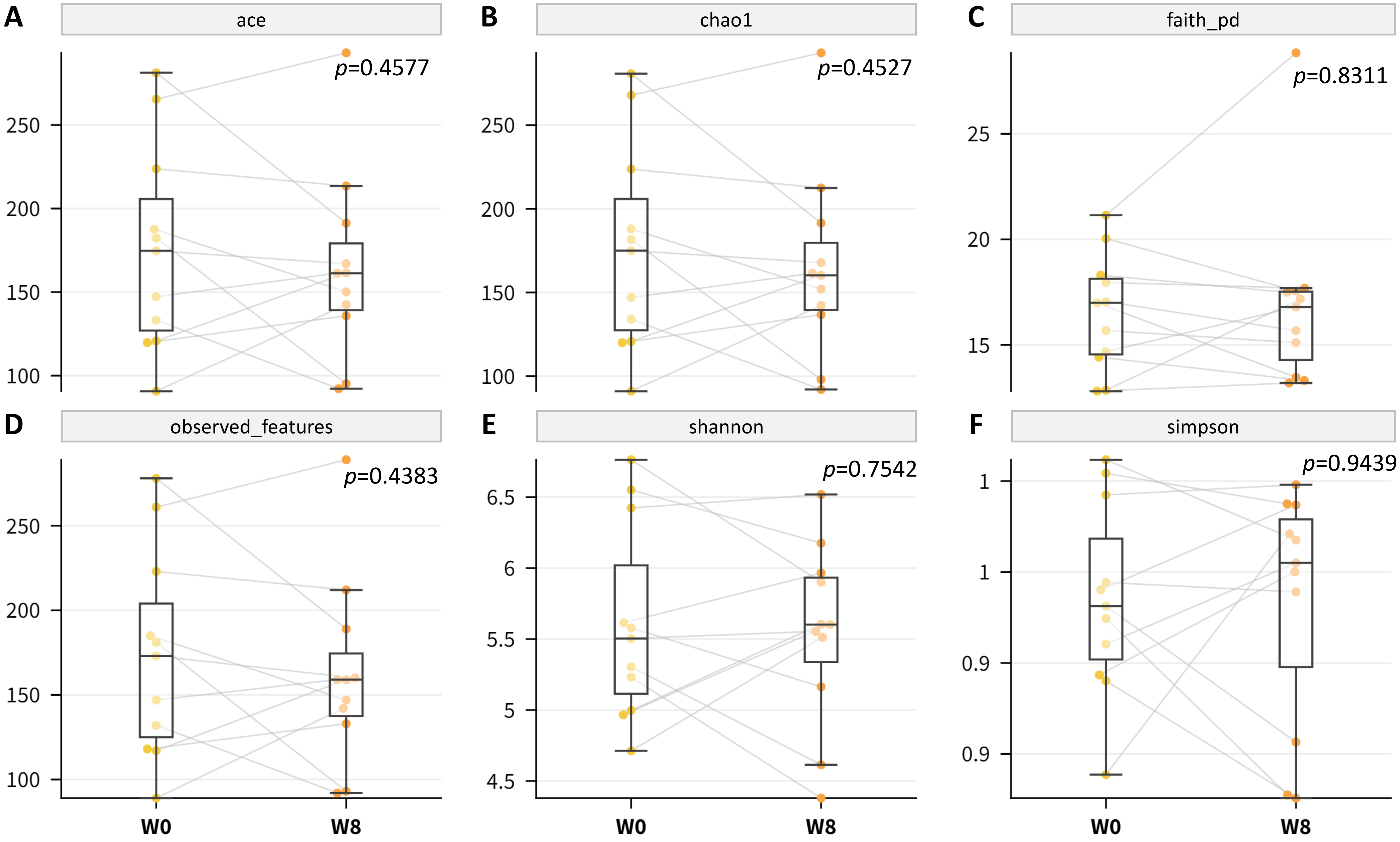

Supplementary Figure S4

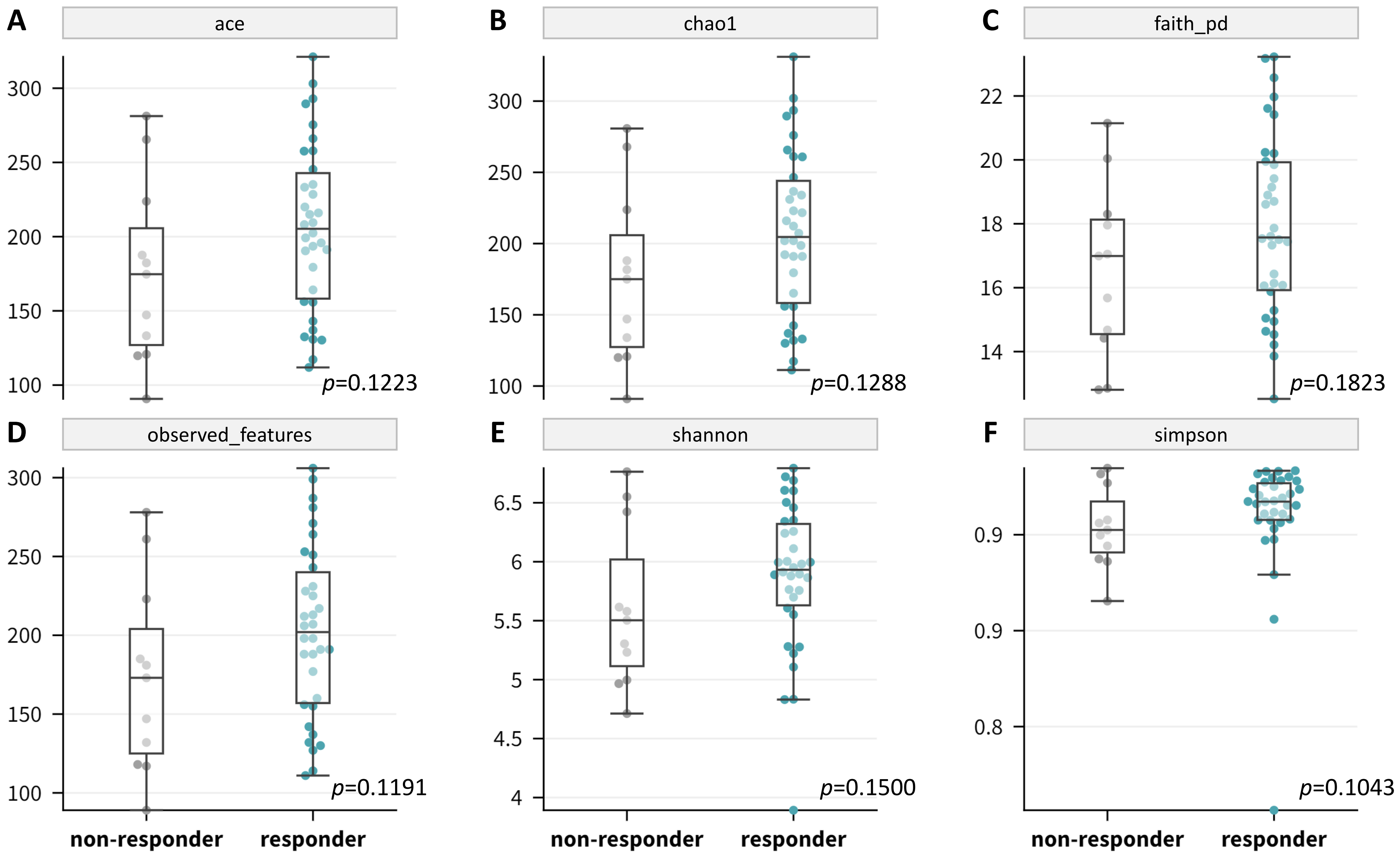

Supplement: Supplementary file 1 [file microorganisms-12-00208-s001.zip › psoriasis_GI_suppl-figures_20231221.pdf]
